# Meeting Report on Experimental Approaches to Evolution and Ecology Using Yeast and Other Model Systems

**DOI:** 10.1534/g3.117.300124

**Published:** 2017-08-08

**Authors:** Daniel F. Jarosz, Aimée M. Dudley

**Affiliations:** * Department of Chemical and Systems Biology and; † Department of Developmental Biology, Stanford University, California 94305 and; ‡ Pacific Northwest Research Institute, Seattle, Washington 98122

**Keywords:** yeast, evolution, ecology, genetics

## Abstract

The fourth EMBO-sponsored conference on Experimental Approaches to Evolution and Ecology Using Yeast and Other Model Systems (https://www.embl.de/training/events/2016/EAE16-01/), was held at the EMBL in Heidelberg, Germany, October 19–23, 2016. The conference was organized by Judith Berman (Tel Aviv University), Maitreya Dunham (University of Washington), Jun-Yi Leu (Academia Sinica), and Lars Steinmetz (EMBL Heidelberg and Stanford University). The meeting attracted ∼120 researchers from 28 countries and covered a wide range of topics in the fields of genetics, evolutionary biology, and ecology, with a unifying focus on yeast as a model system. Attendees enjoyed the Keith Haring-inspired yeast florescence microscopy artwork (Figure 1), a unique feature of the meeting since its inception, and the 1 min flash talks that catalyzed discussions at two vibrant poster sessions. The meeting coincided with the 20th anniversary of the publication describing the sequence of the first eukaryotic genome, *Saccharomyces cerevisiae*. Many of the conference talks focused on important questions about what is contained in the genome, how genomes evolve, and the architecture and behavior of communities of phenotypically and genotypically diverse microorganisms. Here, we summarize highlights of the research talks around these themes. Nearly all presentations focused on novel findings, and we refer the reader to relevant manuscripts that have subsequently been published.

**Figure 1:**
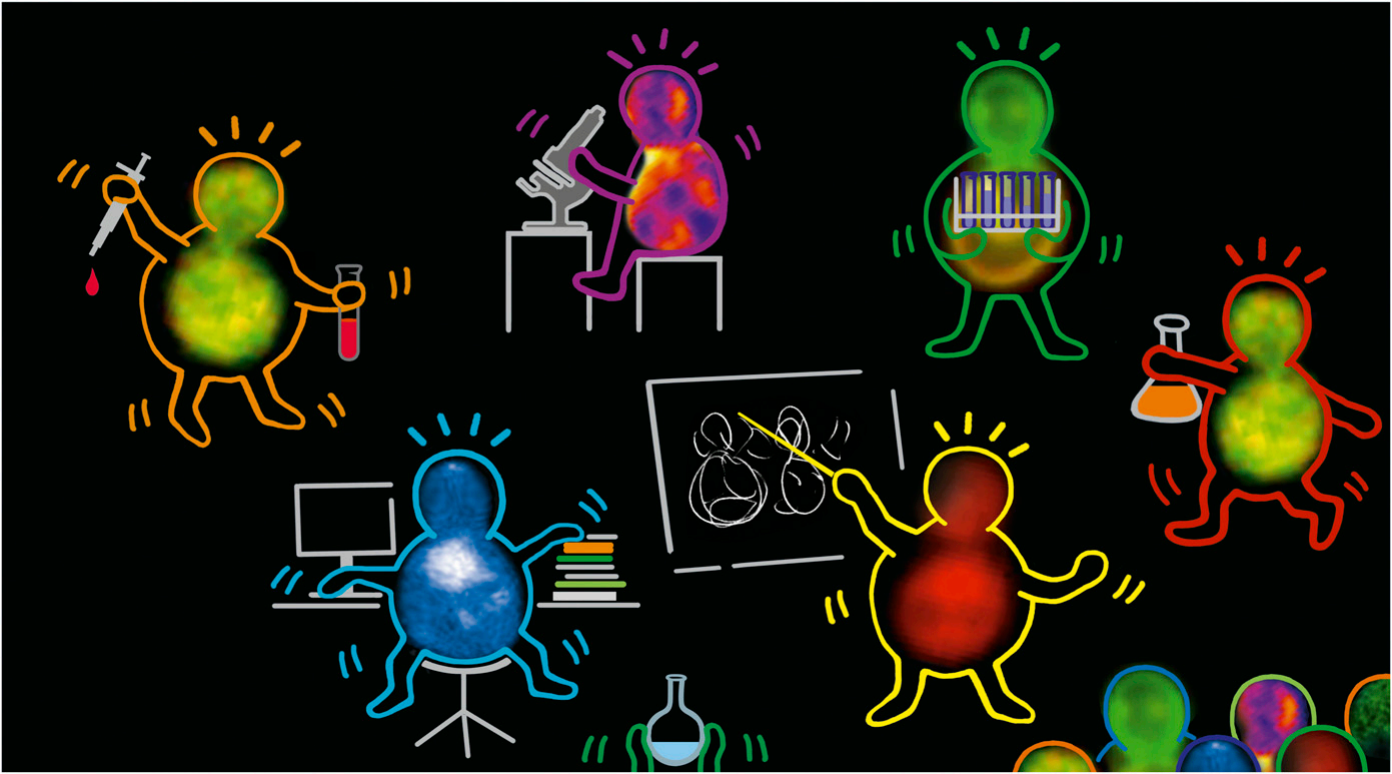
The Keith Haring-inspired artwork using florescence microscopy of yeast cells. Art work by Petra Riedinger and the European Molecular Biology Laboratory. Reproduced with permission.

## Genetics: What is Contained Within the Genome?

The yeast genome project produced a genome assembly of a single strain of *Saccharomyces cerevisiae*, providing the first comprehensive view of the genes that encode its proteins, rRNAs, tRNAs, and small nuclear RNAs (Goffeau *et al.* 1996). Gianni Liti (French National Centre for Scientific Research) presented data from his lab’s efforts to sequence 1002 *S. cerevisiae* strains isolated from the world over, gaining new insights into the evolution, movement, demography, interactions, and phenotypes of this organism. Liti also described the application of long read sequencing technology (PacBio) to 12 strains, which enabled the analysis of structural genome rearrangements (Yue *et al.* 2017). Andrew Murray (Harvard University) focused on the surprising plasticity of one aspect of the genome, the genes essential for the life of the organism. Work from his lab examined this issue by studying how cells suppress the loss or misexpression of essential proteins. This line of investigation raises several interesting questions, such as whether repair trajectories are reproducible and whether certain kinds of mutations (*e.g.*, loss-of-function mutations) are seen more often. The studies provided compelling examples of the power of yeast genetics to tackle challenging and important problems.

Conference coorganizer Lars Steinmetz (Stanford University and EMBL) described his lab’s efforts to understand the diversity of RNA transcripts produced from the genome. Insight has been fueled by the group’s development of a sequencing approach that circularizes cDNAs derived from individual RNA molecules (TIF-seq), thus enabling robust mapping of the 5′ and 3′ ends of individual transcripts. These approaches have revealed an enormous diversity of transcripts, along with rampant bidirectional transcription and antisense regulation (Pelechano *et al.* 2013). Indeed, the diversity of transcripts is so extensive that, for an average gene, ∼10 isoforms account for 80% of the expressed mRNA. Michelle Hays, a graduate student in Harmit Malik’s lab (Fred Hutchinson Cancer Research Center), focused on the topic of genetic conflict between the yeast genome and a potential selfish element, the native 2 micron (2µ) circle. To test whether absence of the 2µ from some natural isolates of *S. cerevisiae* might be the result of a host defense mechanism rather than mere stochastic loss, she developed a GFP-based flow cytometry assay and followed the frequency of 2µ loss through a cross between these strains and a lab strain.

Mapping the relationship between genotype and phenotype has been a major focus of the meeting since its inception. Gaël Yvert (ENS de Lyon) described a new method that permits the mapping of single cell probabilistic traits (Chuffart *et al.* 2016). By acquiring distributions from individual cells and computing pairwise dissimilarities, his lab was able to place individual cells in “phenotypic space.” Performing linkage analysis on these data identified many single cell probabilistic trait loci (scPTL), including some that affected cell shape and expression noise in the yeast galactose regulon.

## Evolution: How do Genomes Evolve?

Much of the research presented at the meeting focused on how genomes evolve, using a wide range of approaches, including ancestral reconstruction, experimental evolution, and even artificial intelligence simulations. One of the meeting’s first speakers, Sarah Otto (University of British Columbia), started the discussion by describing her lab’s efforts to understand epistasis across environments, with specific attention to adaptive mutations. This work highlighted the advantages of yeast as a model to gain information about genetic interactions and the accumulation of incompatibilities among individual mutations (Ono *et al.* 2017).

### By copy number changes

Changes in chromosome number have occurred surprisingly often during the evolution of plants, animals, and fungi. One series of talks focused on the frequency, mechanisms, and consequences of altering chromosome number through polyploidy and interspecies hybridization. Conference coorganizer Jun-Yi Leu (Academia Sinica) focused on the seemingly contradictory observation that while newly-formed polyploid genomes seem intrinsically unstable (often quickly degenerating into aneuploidy or diploidy), for many polyploidization events recorded in evolution, duplicated chromosomes are maintained and genome reorganizations occur much later. Using laboratory evolution experiments to explore this phenomenon, his lab uncovered a molecular mechanism, increased abundance of the Target of Rapamycin Complex 1-activated protein kinase Sch9, which facilitated the maintenance of robust tetraploidy (Lu *et al.* 2016). Anna Selmecki (Creighton University Medical School) focused on how polyploidy influences the rate of evolutionary adaptation by studying isogenic yeast populations that differed only by ploidy (1N, 2N, or 4N). The 4N populations underwent more rapid adaptation, even when the 2N and 4N strains had equal starting fitness. She also discussed examples of mutations that are selectively beneficial in polyploid strains, including whole-chromosome aneuploidy (Selmecki *et al.* 2015).

Meeting coorganizer Maitreya Dunham (University of Washington) presented work investigating hybridization as a mechanism for evolution, and examined the question of what drives changes in genome content, such as loss of heterozygosity. Using experimental studies in chemostats, her lab found that hybrids repeatedly lose heterozygosity at genes that are amplified in the parental genomes and, at least for the examples studied, these events are the result of positive selection of one allele (Smukowski Heil *et al.* 2017). The results imply that even infrequent outcrossing may have lasting impacts on adaptation. Geraldine Butler (University College Dublin) described naturally occurring hybrids in the pathogenic yeast *Candida orthopsilosis* (Schroder *et al.* 2016). Genome sequencing of 27 *C. orthopsilosis* isolates revealed that most were diploid hybrids between two unknown parental species with 5% sequence divergence and were consistent with multiple independent hybridization events between the parental species. Although the parallel emergence of the same hybrid species from multiple independent hybridization events is common in the evolution of fungal plant pathogens, it is less well-documented in human pathogenic fungi.

These initial genome expansion events are often followed by gene reduction and specialization. Giulia Rancati (Institute of Medical Biology) presented work that focused on the impacts of environmental stress on genome instability. Although her research has historically used yeast, Rancati presented work from her lab that used pseudodiploid mammalian cell lines with relatively stable karyotypes and found that short exposures to stresses increased polyploidy and aneuploidy. Brenda Andrews (University of Toronto) presented a study that focused on two possible paths for resolving gene duplication: remaining functionally redundant or acquiring divergent functions. By extending her lab’s well-established system for studying synthetic genetic interactions to the analysis of triple mutants, she found that a high fraction of the triple mutant interactions supported retention of functional redundancy.

### By sexual genome shuffling

Although most microbial evolution studies pragmatically focus on asexual growth in batch cultures or chemostats, in nature meiotic recombination is an important driver of phenotypic diversification. Three speakers described their efforts to develop experimental evolution systems that incorporate recombination. Katy Kao (Texas A&M University) presented her lab’s efforts to construct and evolve a synthetic *Escherichia coli* strain that allows *in situ* genome shuffling by conjugation during adaptation. The results suggested that these “genderless” strains evolved much more rapidly than their asexual counterparts (Peabody *et al.* 2016). Anthony Long (University of California, Irvine) described a yeast synthetic population derived from a four-way cross evolved for 18 wk with meiotic recombination occurring once every 30 mitotic cell divisions. In a lab domestication experiment, adaptation was replicable at the molecular level, primarily due to standing genetic variation, and could be localized to a small number of regions that are a few kilobases in size (Burke *et al.* 2014). These “evolve and resequence” experiments suggest that details of evolution at the molecular level in outbred sexuals may differ from the well-described dynamics of isogenic asexuals. Helen Murphy (College of William and Mary) presented a system to study the evolution of biofilm formation and plastic adherence. In addition to developing a simple method (adherence to a polystyrene bead) to enrich for adherent strains, the experimental design included multiple rounds of selection and meiosis. Over 400 generations of evolution, the ability to adhere to plastic increased over two orders of magnitude, and sexual populations evolved adherence ability more quickly than asexual populations. The majority of the response was due to selection on standing genetic variation present in the initial populations.

### By novel regulatory circuits

Although most transcription factors retain their DNA binding specificity over long evolutionary timescales, regulator–target relationships change rapidly due to the formation and destruction of *cis*-regulatory sequences. Alexander Johnson (University of California, San Francisco) presented the results of one study where his lab found that despite the presence of an unmistakable *GAL4* ortholog in *C. albicans*, the regulation of the highly conserved galactose metabolic pathway is instead driven by the Rtg1 and Rtg3 transcription factors. This is striking because the DNA binding activity of all three transcription factors is identical between *S. cerevisiae* and *C. albicans*, and evidence suggests that regulation by Rtg1 and Rtg3 was the ancestral state (Dalal *et al.* 2016). Thus, the output of a basic circuit has been preserved over evolutionary time, despite the alteration of nearly all of its quantitative and qualitative features. Tyler Starr, a graduate student in Joseph Thornton’s laboratory (University of Chicago), presented work investigating the evolution of DNA binding specificity of the steroid hormone receptors. Ancestral reconstruction has revealed a group of “permissive” substitutions that enabled the historical evolution of novel DNA binding specificity in steroid hormone receptors. Yet whether these substitutions allowed many mutations to accumulate, or just the specific ones that occurred historically, was unknown. Using combinatorial mutagenesis, Starr discovered that although the derived specificity could be achieved in the ancestral background lacking permissive substitutions, all trajectories passed through intermediates with promiscuous DNA binding specificity.

### By the accumulation of adaptive mutations

A great deal of evolutionary theory has been developed to understand the tradeoffs between the diversification enabled by high mutation rates and the associated costs of reduced replication fidelity. Two talks touched on this exciting topic in *E. coli*, where sexual recombination normally cannot subsequently separate the costs of mutator alleles from the benefits of the mutations they may produce. Toon Swings (KU Leuven) presented work investigating adaptation of this bacterium to increasing levels of ethanol stress (Swings *et al.* 2017). As the stress increased, mutator alleles accumulated in the adaptive lineages. With stabilization of the ethanol concentration, these same lineages acquired compensating antimutator alleles. Increased mortality was a critical cost of hypermutation, and survival increased significantly when the final mutation rate was reduced. Thomas Ferenci (University of Sydney) presented work from his lab characterizing mutations in six nutritional states. Looking at the *cycA* reporter gene in carbon and phosphate limitation revealed not only significant increases in mutation rates, but also changes in the spectrum of mutations produced and selected during experimental evolution (Maharjan and Ferenci 2017). Taken together, these results may motivate a reconsideration of stress-induced mutagenesis.

Dmitri Petrov (Stanford University) described state-of-the-art barcoding technology that allowed his lab, in collaboration with those of Gavin Sherlock and Daniel Fisher, to observe the spread of thousands of individual adapting lineages in a serial dilution culture system. These data provided comprehensive and systematic measurements of the distribution of rates and strengths of selective benefit of practically all single adaptive mutations that drove early adaptation. The experimental design allowed the recovery and identification of hundreds of individual adaptive mutations, many of which turned out to be in the Ras/PKA and Tor pathways (Venkataram *et al.* 2016). Gregory Lang (Lehigh University) presented work investigating the mutations responsible for long-term adaptive evolution in clones isolated from independent lineages of experimentally evolved yeast. Consistent with theory, most mutations in these genomes were neutral, with a handful of driver mutations that were present in most clones (Buskirk *et al.* 2017).

Christoph Adami (Michigan State University) presented a series of evolutionary simulations with digital “individuals” to investigate the influence of mutation rate, population size, genetic architecture, and structure of the fitness landscape on adaptive evolution. His lab used this approach to investigate why small populations appear to be more robust to genetic drift. The simulations suggest that strong drift allows populations of different sizes to evolve similar complexity through different trajectories, with small populations evolving larger genomes through the fixation of slightly deleterious insertions and large populations using rare beneficial insertions (LaBar and Adami 2016).

## Ecology: How do Individuals Interact?

Living in a community is an emergent property of life; interaction between individuals and their environment gives rise to complex behaviors. While researchers often simplify their thinking about unicellular microorganisms by considering them individually, in actuality microorganisms live in communities of near clonal individuals, genetically distinct individuals of the same species, mixed populations of different species, or microbial–host environments. A number of talks explored the frontiers of microbial ecology at these different scales.

### Between diverse cell types

One series of talks focused on how yeast differentiate into distinct cell types. Zdena Palková (Charles University in Prague) presented collaborative work between her lab and that of Libuše Váchová (Institute of Microbiology of the CAS) on the development and differentiation of yeast colonies. Recently, their labs discovered two spatially organized cell types in yeast colonies: respiratory competent L cells in the lower regions that provide nutrients to the U cells in the upper regions that have metabolic properties important for longevity and response to starvation. Using florescent reporters for protein expression specific to these cell types has uncovered new information about the spatial organization of the retrograde signaling pathway and the role of mitochondria in communities of cells growing in colonies (Podholova *et al.* 2016). Paul Magwene (Duke University) discussed how genetic background influences a yeast cell’s response to starvation cues, which may include continuing mitotic growth, switching to pseudohyphal growth, or undergoing sporulation. His lab found that these responses can be influenced by natural variation in cAMP/PKA signaling, *FLO11* and its regulator *MIT1*, and several other loci. Zoran Marinkovic, a student in the labs of Pascal Hersen (University of Paris Diderot/CNRS) and Ariel Lindner (INSERM), described a microfluidic device for visualizing the dynamics of cells growing in multi-layered colonies by time-lapse microscopy. Marinkovic used this technique to examine growth and gene expression patterns in colonies, focusing on the Hxt1-7 hexose transporters as a means of reconstructing the glucose concentration gradient in the colony at high spatiotemporal resolution.

Wenying Shou (Fred Hutchinson Cancer Research Center) presented work from her lab investigating evolutionary changes in a synthetic yeast cooperative community, where two strains each pay a fitness cost to supply the other with an essential metabolite (lysine or adenine). In a spatially-structured environment, cheaters (low-releasers) were physically excluded from cooperators (releasers) and failed to grow to high levels (Momeni *et al.* 2013). In a well-mixed environment, both strains evolved to grow better in the metabolite-limited community, and were expected to become cheaters. Even though one strain evolved to cheat, the other strain rapidly evolved increased release rate on a per-cell basis.

### Between different species

Outside of the laboratory, yeasts are commonly found associated with a wide array of other organisms, and several talks explored these interspecies interactions. Kiran Patil (EMBL Heidelberg) described his lab’s work investigating interactions between organisms that commonly cooccur in nature, *S. cerevisiae* and lactic acid bacteria. Results from metabolomics, modeling, and genetic approaches established that yeast stably supports the growth of symbiotic lactic acid bacteria through nitrogen overflow, resulting in the active secretion of specific amino acids. Catrin Günther (University of Lincoln) examined the effect of various fruit substrates on interactions between yeast and *Drosophila*, with a focus on whether particular yeast genotypes are differentially attractive to *Drosophila simulans* and *D. melanogaster*. The results demonstrated that the attraction of *D. simulans* to various *S. cerevisiae* strains is heavily dependent upon the fruit context, whereas *D. melanogaster* appears to be more universally attracted to *S. cerevisiae*. The observed divergence in chemosensory preference for apples naturally infected with microbes suggests spatiotemporal variation in the abundance of these sympatric species and a likely mechanism for coexistence in an orchard environment.

### Between fungal pathogens and the mammalian host

One class of interspecies interactions that are of intense interest are the interactions between fungal pathogens and mammalian hosts. A series of talks addressed this topic from several angles. One major goal of understanding pathogenic fungi is to combat infections, for which resistance (including multi-drug resistance) is an increasing problem. Jane Usher (University of Exeter) presented results from a study that leveraged the *S. cerevisiae* deletion library to identify genetic interactions with highly fluconazole-resistant gain-of-function mutants in the Pdr1 transcription factor from *C. glabrata*. The screen identified components of the SAGA transcriptional coactivator complex as potential targets for combating drug resistance.

Several talks addressed genotypic and phenotypic heterogeneity, an issue that has long been appreciated, but only recently become tractable to study in pathogenic fungi. Anja Forche (Bowdoin College) focused on the generation of genotypic diversity in *C. albicans* following passage through mouse models of oral and systemic infection. A variety of methods, including flow cytometry and double digest RAD sequencing, detected numerous genomic changes. Interestingly, distinct changes were seen in different infection models, *e.g.*, enrichment of chromosome 6 trisomies in the oral model, raising important questions about selective pressures in different host niches. Conference coorganizer Judith Berman (Tel Aviv University) focused on phenotypic diversity. Populations of pathogenic fungi exhibit various mechanisms of resistance or tolerance to antifungal drugs, ranging from resistance (in which all members of the population grow at high drug concentrations) to persistence (in which only a few members of the population grow at high drug concentrations). Clinical assays currently ignore these population distributions, which can be visualized as breakthrough growth within a general zone of growth inhibition. Characterizing the type of resistance, including heteroresistance, could have important implications for combating different types of recalcitrant infections. Gilad Yaakov (Weizmann Institute of Science) discussed phenotypic persistence in *S. cerevisiae*. The study identified a subpopulation of persister cells that arose during antifungal drug treatment, and subsequently demonstrated that the persister phenotype was triggered by spontaneous, long-lived DNA damage that induces the general stress response. These preadapted persisters better survive extreme stresses and drugs, at the expense of slower growth in nonstress conditions. In addition, DNA-damaged persisters are enriched for random mutations, resulting in increased genetic diversity among the relatively small number of survivors from a severely stressed population (Yaakov *et al.* 2017).

## Technology: Investigating Longstanding Questions with High Throughput Methods

The recent explosion of technological innovation has sparked interest in addressing longstanding questions on scales not previously possible. Fyodor Kondrashov (Centre for Genomic Regulation) presented work from his lab that examined how individual proteins evolve by quantitatively assaying the fluorescence of 55,000 GFP mutants in *E. coli* (Sarkisyan *et al.* 2016). Because most clones contained multiple mutations, nonlinear interactions (epistasis) between mutations in the same clone could be detected and negative epistasis was common. Analogous experiments with *HIS3* in yeast revealed that the same variants that destroy activity in one organism were often permitted in other related organisms. Yitzhak Pilpel (Weizmann Institute of Science) presented work (Frumkin *et al.* 2017) that extends his lab’s longstanding interest in how cells minimize the cost of protein expression. Using a library of 14,000 related gene variants fused to sfGFP, his lab investigated several possible ways to limit the cost of gene expression. Sequencing these libraries after outgrowth revealed that clones producing more RNA than protein are less fit than variants with low mRNA production and more efficient translation. The analysis further suggested that high-fitness variants utilizing amino acids that are cheap to synthesize and that are less hydrophobic possessed mechanisms for slowing down the ribosome early in elongation. Similar signatures were observed in natural *E. coli* genes, providing a window into design elements that optimize the economy of protein expression.

Overall, the questions and results discussed at the meeting provided stimulating and thought-provoking insights into evolution and ecology. Twenty years ago the scientists who sequenced the yeast genome noted that, “New graduate students are already wondering how we all managed in the “dark ages” before the sequence was completed...” and remarked that the larger task of understanding the function and evolution of the genes identified would “require a worldwide effort” (Goffeau *et al.* 1996). This meeting highlighted some of the most interesting and creative research in the fields of evolution and ecology worldwide, much of which was made possible by the yeast genome project and technologies it subsequently enabled.

The conference will be held again in Heidelberg, Germany on October 17–20, 2018.
